# Psychological Disorders, Cognitive Dysfunction and Quality of Life in Nasopharyngeal Carcinoma Patients with Radiation-Induced Brain Injury

**DOI:** 10.1371/journal.pone.0036529

**Published:** 2012-06-11

**Authors:** Yamei Tang, Donghua Luo, Xiaoming Rong, Xiaolei Shi, Ying Peng

**Affiliations:** 1 Department of Neurology, Sun Yat-sen Memorial Hospital, Sun Yat-sen University, Guangzhou, China; 2 Key Laboratory of Malignant Tumor Gene Regulation and Target Therapy of Guangdong Higher Education Institutes, Sun Yat-sen University, Guangzhou, China; 3 Department of Nasopharyngeal Carcinoma, Cancer Center of Sun Yat-sen University, Guangzhou, China; Johns Hopkins Hospital, United States of America

## Abstract

**Purpose:**

To evaluate factors affecting psychology, cognitive function and quality of life (QOL) of nasopharyngeal carcinoma (NPC) patients with radiation-induced brain injury (RI).

**Methods and Materials:**

46 recurrence-free NPC patients with RI and 46 matched control patients without RI were recruited in our study. Subjective and objective symptoms of RI were evaluated with the LENT/SOMA systems. Psychological assessment was measured with Self-Rating Anxiety Scale (SAS) and Self-Rating Depression Scale (SDS). Montreal Cognitive Assessment (MoCA) was carried out in these patients for assessing their cognitive function. QOL was evaluated by means of WHOQOL BREF.

**Results:**

Of the patients with RI, 39(84.8%) had depression and 40(87.0%) had anxiety. The patients with RI got higher scores both in SDS and SAS than those without RI (SDS, 63.48±8.11vs. 58.67±7.52, p = 0.008; SAS, 67.36±10.41vs. 60.34±9.76, p = 0.005). Score in MoCA of patients with RI was significantly lower than that of patients without RI (21.32±2.45vs. 25.98±1.73, p<0.001). SAS was positive correlated with post-radiotherapy interval. Both SAS and SDS had a significantly positive correlation with the rank of SOMA, while MoCA had a significantly negative correlation with SOMA. Chemotherapy was a risk factor for cognitive dysfunction. In addition, patients with RI got significantly lower scores in physical health (16.50±11.05 vs. 35.02±10.43, p<0.001), psychological health (17.70±10.33 vs. 39.48±12.00, p<0.001) and social relationship (48.00±18.65 vs. 67.15±19.70, p<0.001) compared with those in patients without RI. Multiple linear regression analysis revealed that anxiety and cognitive impairment were significant predictors of global QOL.

**Conclusions:**

NPC patients with RI exhibit negative emotions, impaired cognitive function and QOL. The severity of clinical symptoms of RI plays an important role in both emotions and cognitive function. Anxiety and cognitive impairment are associated with decreased QOL.

## Introduction

Nasopharyngeal carcinoma (NPC) is known as the high incidence cancer in China [1], especially in Guangdong Province. Radiotherapy (RT) is a long-standing mainstay of NPC treatment. The cancer-specific survival rate of NPC is generally favorable, and thus long term side effects of treatment are of concern in survivors. Among the large range of complications encountered, radiation-induced brain injury (RI) is a severe complication.

**Figure 1:**
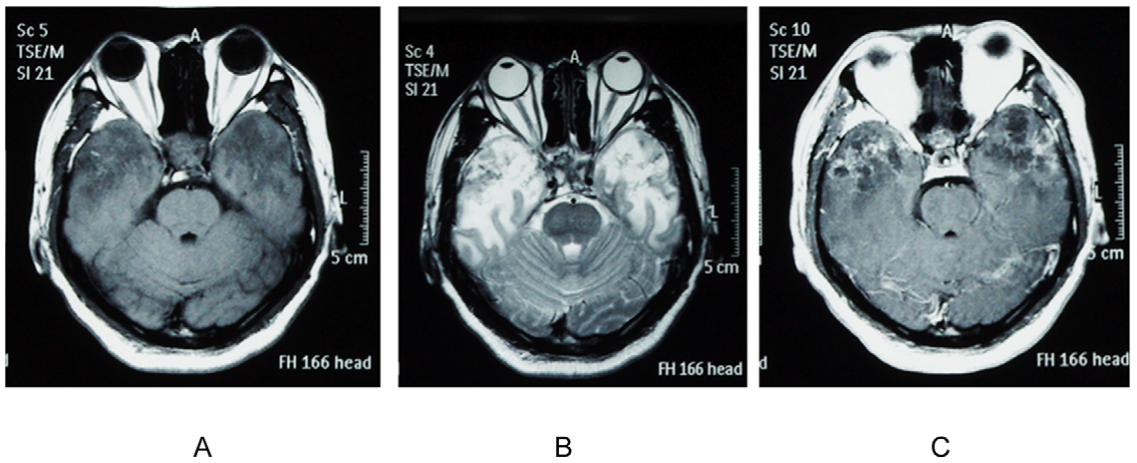
The brain MRI scan of a patient with RI. (A) The axial T1-weighted imaging showed relatively low-signal-intensity lesions in the bilateral temporal lobes. (B) The T2-weighted imaging revealed high-signal-intensity lesions in the bilateral temporal lobes. T1-weighted image after contrast administration (C) showed irregular edge contrast enhancement of the bilateral temporal lobes.

Compared with patients who had tumors in other head and neck regions, patients with NPC had much worse impairment in social and role function [2], [3]. Although there have been some reports [4], [5]about psychological disorders, cognitive dysfunction and QOL of NPC patients following RT, most of them focused on the effects of therapies and with a relatively limited post-radiotherapy interval. The psychological disorders and QOL of patients with a long post- RT interval, especially of patients with RI are seldom addressed. Comparison of psychological disorders and QOL between patients with and without RI is not fully elucidative. Whether psychological disorders are the complications of RI or just frequently observed in patients following RT is still poorly known. For these reasons, we undertook a psychological study (including SAS and SDS), cognitive (MoCA) and QOL (WHOQOL BREF) assessment in NPC patients with RI. The results were compared with those of a matching post-radiotherapy (post-RT) NPC patients without RI.

## Methods

This project was approved by an authorized human research review board in our institute (Ethics Committee of The Sun Yat-sen University). Patients included in this study were inpatients and outpatients of the Sun Yat-sen Memorial Hospital of Sun Yat-sen University and the Cancer Center of Sun Yat-sen University. Written informed consents were obtained from all involved subjects.

### Patients

Between February 2009 and March 2010, patients who fulfilled the following eligibility criteria were recruited as case group: (1) a history of NPC with RT; (2)the clinical manifestation and the magnetic resonance imaging (MRI) (Figure 1) or computed tomography (CT) scan met the diagnosis of RI in Merritt's Neurology(10*^th^* edition). All patients had clinical symptoms of RI. (3) no evidence of symptomatic recurrent tumor, brain metastasis, brain abscess, any intracranial tumor, cerebral infarction, demyelinating disease, encephalitis or other central nervous system diseases; (4) no evidence of disturbance of consciousness or unstable vital signs. There were 46 patients matching the criteria including 35 males and 11 females.

**Table 1:** Characteristics of the two groups.

Characteristics	Number of patients (%)	
	Case group (n = 46)	Control group (n = 46)
Gender		
men	35(76.1)	35(76.1)
women	11(23.9)	11(23.9)
	P>0.05	
Age(ys) Mean ± SD	39.8±15.1	39.6±14.6
	p>0.05	
Educational level		
illiteracy	2(4.3)	2(4.3)
Primary school	9(19.6)	9(19.6)
Junior high school	17(37.0)	16(34.8)
Technical secondary school Or Senior high school	15(32.6)	17(37.0)
Undergraduate course	3(6.5)	2(4.4)
Postgraduate above	0	0
	p>0.05	
Residential place		
City	20(43.5)	19(41.3)
Small town	19(41.3)	19(41.3)
Countryside	7(15.2)	8(17.4)
	p>0.05	
Post-RT(ys)	6.0±3.5	5.7±3.1
	p>0.05	
Chemotherapy	12(26.1)	12(26.1)
	p>0.05	

*Abbreviation*: SD  =  standard deviation.

The same amount of subjects was recruited within the same period as control group. The subjects were matched for age, gender, educational level, treatment modalities, and post-RT interval. They also followed the criteria mentioned above except the second one.

### Methods to collect the historical information

The following data were retrieved from the clinical notes: (1) age, gender, education background, occupation, marriage, residence area, medical information (date of starting RT, dosage, the target volume, the duration time, with or without chemotherapy, whether suffering from another central nervous system diseases); (2) physical examination findings; (3) auxiliary examinations including brain CT or MRI scan; (4) radiation toxicities scores assessed by the Late Effects of Normal Tissue (LENT) – Subjective, Objective, Management, Analytic (SOMA) Scales [6] in patients with RI.

**Table 2:** Depression and Anxiety in Two Groups.

	Case group (n = 46)	Control group (n = 46)	P value
Morbidity of depression			
Depression (%)	39(84.8)	36(78.3)	0.420
No depression (%)	7(15.2)	10(22.7)	
SDS score	63.48±8.11	58.67±7.52	***0.008***
Morbidity of anxiety			
Anxiety (%)	40(87.0)	38(82.6)	0.562
No anxiety (%)	6(13.0)	8(17.4)	
SAS score	67.36±10.41	60.34±9.76	***0.005***

SDS score and SAS score are presented as mean ± standard deviation.

*Abbreviations*: SDS  =  Self-rating Depression Scale; SAS  =  Self-rating Anxiety Scale.

### Neuropsychological test

*Self-Rating Depression Scale (SDS):* It is a 20-item self-reported measurement of the symptoms of depression that includes statements about cognitive, somatic, psychomotor, and affective symptoms. Each item is scored from 1 to 4. Raw score is converted into standardized score. A cut-off higher than 53 was used to define presence of depression according to the Chinese version of this scale [7].

*Self-Rating Anxiety Scale (SAS):* SAS is a 20-item scale, with some of the items keyed positively and some negatively. They are answered on a four-point scale ranging from 1(none or a little of the time) to 4(most or all of the time). After being converted into the standardized score, a cut-off 50 was used to define anxiety according to the Chinese version of the scale [7].

*Montreal Cognitive Assessment (MoCA):* It assesses different cognitive domains: attention and concentration, executive functions, memory, language, visuoconstructional skills, conceptual thinking, calculations and orientation. Time to administer the MoCA is approximately 10 minutes. The total possible score is 30, a score of 26 or above is considered normal.

*WHOQOL-BREF:* The WHOQOL-BREF instrument comprises 26 items, which measure the following broad domains: physical health, psychological health, social relationships, and environment [8].

**Table 3:** Multiple linear regression analysis of age, gender, education, post-RT interval, and chemotherapy to SAS.

Model	B-coefficient	Std. Error	P value	Adj. R2
1 (constant)	59.834	2.239	<0.001	
Post-RT interval	0.687	0.334	0.043	0.034

*Abbreviations*: SAS  =  Self-rating Anxiety Scale. Post-RT interval  =  post-radiotherapy interval; Adj.  =  adjusted; Std.  =  standard.

**Table 4:** Excluded variables^b^.

Model	Beta In	t	P value	Partial correlation	Collinearity Statistics
					Tolerance
1 age	−0.010^a^	−0.090	0.929	−0.010	0.945
gender	0.34^a^	0.323	0.748	0.034	0.963
chemotherapy	0.067^a^	0.650	0.518	0.069	1.000
Education- background	0.029^a^	0.279	0.781	0.030	0.998

^a^ Predictors in the Model: (constant), post-RT interval.

^b^ Dependent Variable: SAS.

### Statistical analysis

Paired-samples t test was used to compare the clinical characteristics and the scores of SDS, SAS, MoCA and QOL between the case group and control group. χ2 test was performed to compare the depression and anxiety scores between the two groups. Stepwise multiple linear regression was applied to explore predictors of psychological and cognitive disorders. Spearman's correlation was performed to examine the relationship between SOMA and the scores of SDS, SAS, MoCA. All tests were two-tailed and a 5% significance level was used for statistical significance. The SPSS for windows, version 13.0 was used for data processing.

## Results

In case group, 46 patients were included in the analysis. The median time after RT was 6.0±3.5 years (S.D.) (range from 1 to 19 years). The accumulated radiation doses were 68 to 76 Gy (median, 70.2 Gy), with 2 Gy per fraction applied to the primary tumor, and the estimated maximal dose to the adjacent brain was 70 Gy–73 Gy. All patients were treated with one fraction daily for five days per week. Nineteen of them suffered from hypertension, diabetes, chronic bronchitis and other medical morbidities. Sixteen of them received chemotherapy (thirteen patients received concurrent chemotherapy, three patients received both neoadjuvant and concurrent chemotherapy). Forty-six matched controlled post-RT patients without RI were recruited as control group. Two major radiation fields, facial-cervical fields and facial-cervical split fields were used in these patients. The demographic and other background data for the two groups were similar (Table 1).

**Table 5:** Multiple linear regression analysis of age, gender, education, post-RT interval, and chemotherapy to MoCA.

Model	B-coefficient	Std. Error	P value	Adj. R2
1 (constant)	21.050	1.157	<0.001	
chemotherapy	1.575	0.673	0.022	0.047

*Abbreviations*: MoCA  =  Montreal Cognitive Assessment; Adj.  =  adjusted; Std.  =  standard.

**Table 6:** Excluded variables^b^.

Model	Beta In	t	P value	Partial correlation	Collinearity Statistics
					Tolerance
1 age	−0.083^a^	−0.769	0.444	−0.081	0.907
gender	0.135^a^	1.319	0.191	0.138	0.993
Post-RT interval	−0.012^a^	−0.119	0.905	−0.013	1.000
Education- background	0.033^a^	0.324	0.747	0.034	0.993

^a^ Predictors in the Model: (Constant), chemotherapy.

^b^ Dependent Variable: MOCA.

### Psychopathology characteristics between the two groups

In case group, 39(84.8%) patients had depression, 40(87.0%) had anxiety, and 36 (78.3%) had both. In control group, 36 (78.3%) patients had depression, 38(82.6%) had anxiety. Table 2 shows that the overall incidence of depression and anxiety are not significantly different between the two groups (84.8% vs. 78.3%, P = 0.420; 87.0% vs. 82.6%, P = 0.562). But the standardized SDS score and SAS score were much higher in the case group than those in the control group (63.48±8.11 vs. 58.67±7.52, P = 0.008; 67.36±10.41 vs. 60.34±9.76, P = 0.005).

**Figure 2:**
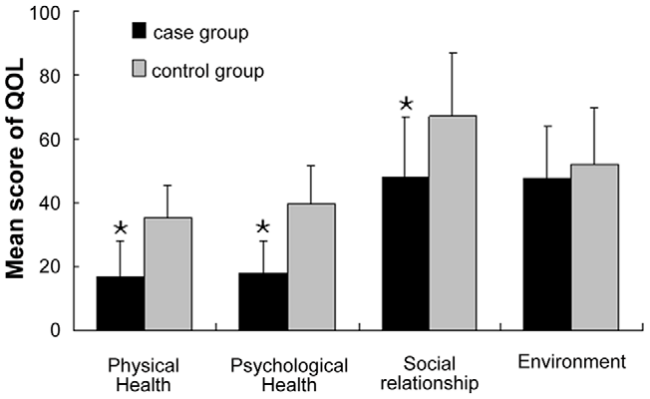
QOL in two groups. The bars represented four domains of QOL as mean score ± standard deviation. Patients in case group got significantly lower score in the physical health (p<0.001), psychological health (p<0.001) and social relationship (p<0.001). There was no significant difference in score of environment domain between two groups (p = 0.203). Abbreviations: QOL  =  quality of life.

According to the categorical fashion, the incidence of severe depression in the case group was 15.2%(seven patients). In the control group, none suffered from severe depression. The percentage of patients with severe depression as defined by standardized SDS, was significantly higher in the case group compared with the control group (p = 0.017).

In the case group, the number of patients with severe anxiety was 25 (54.3%). While in the control group, the number of severe anxiety was 9(19.6%). The percentage of patients with severe anxiety was significantly higher in the case group (p = 0.004).

### Cognitive function in the two groups

The MoCA score of case group and control group was (21.32±2.45) and (25.98±1.73) respectively. Patients without RI tended to score higher than those with RI (p<0.001).

### Determinants of SDS, SAS and MoCA

A series of stepwise linear regression analyses was performed. Results showed that post-RT was the significant predictor of SAS. SAS score was significantly positive correlated with post-RT interval (p = 0.043) (Table 3, Table 4). Besides, chemotherapy was the significant predictor of MoCA (P = 0.047) (Table 5, Table 6). But, gender, education background, age, post-RT interval or chemotherapy had no significant association with severity of SDS (p>0.05).

**Table 7:** Multiple linear regression analysis of age, gender, education, post-RT interval, chemotherapy, SAS, SDS and MoCA to predict QOL.

Model	B-coefficient	Std. Error	P value	Adj. R2
1 (constant)	−6.532	29.663	0.826	
MoCA	7.109	1.243	<0.001	0.258
2 (constant)	52.892	40.911	0.199	
MoCA	6.650	1.241	<0.001	
SAS	−0.760	0.367	0.041	0.284

*Abbreviations*: SAS  =  Self-rating Anxiety Scale; SDS  =  Self-rating Depression Scale; MoCA  =  Montreal Cognitive Assessment; QOL  =  quality of life; Adj.  =  adjusted; Std.  =  standard.

**Table 8:** Excluded variables^b^.

Model	Beta In	t	P value	Partial correlation	Collinearity Statistics
					Tolerance
1 age	0.027^a^	0.295	0.768	0.031	1.000
gender	−0.109^a^	−1.198	0.234	−0.126	0.987
Post-RT interval	−0.063^a^	−0.699	0.486	−0.074	1.000
chemotherapy	−0.050^a^	−0.534	0.595	−0.057	0.943
Education-background	−0.077^a^	−0.856	0.394	−0.090	1.000
SAS	−0.186^a^	−2.069	0.041	−0.214	0.968
SDS	−0.098^a^	−1.064	0.290	−0.112	0.954
2 age	0.016^b^	0.177	0.860	0.019	0.996
gender	−0.092^b^	−1.023	0.309	−0.108	0.978
Post-RT interval	−0.026^b^	−0.280	0.781	−0.030	0.955
chemotherapy	−0.029^b^	−0.316	0.753	−0.034	0.931
Education- background	−0.074^b^	−0.828	0.410	−0.088	0.999
SDS	−0.081^b^	−0.891	0.375	−0.095	0.946

^a^ Predictors in the Model: (Constant), MOCA.

^b^ Predictors in the Model: (Constant), MOCA, SAS.

LENT/SOMA scale was used to evaluate the severity of clinical symptoms of RI. Correlation analysis demonstrated that SAS and SDS were significantly positively correlated with SOMA (Spearman's correlation coefficient  = 0.335, p = 0.023; correlation coefficient  = 0.299, p = 0.044). Also, cognitive function was significantly negatively correlated with SOMA (Spearman's correlation coefficient  = −0.472, p = 0.001).

### WHOQOL-BREF

The raw scores are transformed into standard scores in line with the WHOOL-100 Instrument [9]. The higher the score, the better QOL the patients felt. Comparison of QOL scores between two groups presented in Figure 2. Patients in the case group got significant lower score in physical health compared with that in the control group (16.50±11.05 vs. 35.02±10.43, p<0.001). The mean score for psychological health of case group was 17.70±10.33, while the score of control group was 39.48±12.00, there was a significant difference between two groups (p<0.001). Also in social relationship, the score in case group and control group was 48.00±18.65 and 67.15±19.70 respectively, the difference is significant (p<0.001). Yet, in environment domain, the score in two groups was similar (47.39±16.69 vs. 52.00±17.74, p = 0.203).

To identify the determinants of QOL, the demographic data and scores of SAS/SDS/MoCA were entered into the regression analysis. We found that SAS score (p = 0.041) and MoCA score (p<0.001) were both the significant predictors (Table 7, Table 8).

## Discussion

This study investigates emotional status, cognitive function and QOL of post-RT NPC patients complicated with RI. Data were compared with those of post-RT NPC patients without RI. According to SDS and SAS assessment, more than three fourths of patients after RT had either depression or anxiety. Previous studies suggested that psychological disorders such as depression and anxiety were apparent as early as the start of RT, and might remain throughout the treatment [10], [11], [12]. Lee [13] carried out a prospective study of the impact of RT on the psychosocial condition of NPC patients. The results indicated that the period from diagnosis to 2-month post RT was a high-risk period emotionally. After treatment, most patients showed resilience and resumed their pretreatment level of functioning by the end of the year. The post-RT interval in our study was 6.0±3.5 years in the case group and 5.7±3.1 years in the control group, which indicated that psychological problems lasted long after radiotherapy. Yet, how these psychological disorders develop or how they influence patients' QOL when patients suffer from RI remains unclear. In our study, depression and anxiety incidence in patients with RI was similar to that of patients without RI. But according to the SDS and SAS scores, depression and anxiety were more severe in patients with RI than those in patients without RI. These indicated that RI itself may aggravate the severity of depression or anxiety. In regard to the factors influencing anxiety and depression, we found that except for post-RT interval, age, gender, education and chemotherapy had no significant correlation with either anxiety or depression. The major difference between the case group and control group were the clinical complications caused by radiotherapy. LENT/SOMA, a tool to evaluate the severity of brain complication, was proved to had correlation with both SAS and SDS, which suggested that RI was likely to aggravate the severity of psychological disorders. It is reasonable that patients feel upset when they still have to confront RI which is unexpected even unbearable after the difficult experience of RT. Patients often pay close attention to their slight changes of body, worry about the recurrence of tumors, and keep on consulting doctor frequently. All these are typical behaviors of anxiety.

MoCA assesses different cognitive domains: attention and concentration, executive functions, memory, language, visuoconstructional skills, conceptual thinking, calculations, and orientation. Previous study found that the late effects of RT on cognitive function included three situations: transitory cognitive impairment primarily affecting attention and recent memory, which usually occurred within the first 6 months after cranial RT; mild or moderate cognitive impairment and dementia with leukoencephalopathy occurred in the late delayed period [14]. Compared to dementia, mild to moderate cognitive dysfunction is much more frequent in long-term survivors. In our study, the patients had worse cognitive function than patients without RI. This result was consistent with earlier studies [12], [15], [16]. From these studies, there appeared to be a correlation between the severity of cognitive deficits and severity of abnormalities of white matter or temporal lobe radio-necrosis. As cognitive dysfunction may result from multifactorial complex interactions, including preexisting cognitive abnormalities, concomitant treatments (chemotherapy, antiepileptic, psychotropic drugs), match case control study can avoid the confounding factors. From our results, chemotherapy was demonstrated to be predictor of cognitive dysfunction. The combination of RT and chemotherapy increased the incidence of dementia have been proved by early studies [17]. Through match control, usage of chemotherapy was similar in the two groups, therefore we thought that impaired cognitive function was mostly due to RI. Damage to cerebral blood vessels based on the radiosensitivity of endothelium, as well as vascular vulnerability to RT lead to RI and some represent cognitive impairment.

In our study, the most common symptoms in patients with RI included impaired cognition, bulbar palsy, headache, dizziness, syncope. Bulbar palsy is often caused by injury to the brain stem or the lower cranial nerves, and it may eventually develop dysphagia [18], [19], which significantly decrease patients' quality of life. Patients with NPC after radiotherapy often have radiation-induced lesions in the temporal lobe and therefore manifest significant impairment in memory, language, motor performance, and executive function [20]. If the lesions aggravate, or superimpose with while matter edema, the patient might have headache, dizziness and severe cognitive impairmen [21] Besides, unusual complications such as oscillopsia [22], vertigo [23] are also reported in long-term NPC survivors. All the above symptoms decrease quality of life in NPC patients inevitably. Our score of QOL showed a significant difference between patients with RI and patients without RI in the following domains: physical health, psychological health and social relationships. These results support our presumption. Regression analysis also revealed that anxiety and cognitive impairment might explain their lower score of QOL. It is similar to the other studies [4], [24], [25] that, emotional status, including depression and anxiety, are likely to impair QOL.
